# Strongyloides stercoralis infection in a DLBCL patient treated with rituximab and BTK inhibitor: A case report and literature review

**DOI:** 10.1097/MD.0000000000041533

**Published:** 2025-02-21

**Authors:** Wanyi Liu, Zechuan Wang, Huiqiang Wu, Lili Zeng, Nina Cai, Weihuang Zhuang, Jianxin Guo

**Affiliations:** a Department of Hematology, The Second Affiliated Hospital, Fujian Medical University, Quanzhou, Fujian Province, China; b The Second Affiliated Hospital of Fujian Medical University, Quanzhou, Fujian Province, China; c The Second Clinical Medical College, Fujian Medical University, Fujian, China.

**Keywords:** diffuse large B-cell lymphoma, parasitic infection, targeted therapy

## Abstract

**Rationale:**

Diffuse large B-cell lymphoma (DLBCL) is a common subtype of non-Hodgkin’s lymphoma characterized by high malignancy, rapid onset and aggressive clinical behavior. The disease exhibits considerable heterogeneity, which influences clinical and immunophenotypic characteristics, which in turn affect treatment outcomes and prognosis. Recently, targeted therapies have been introduced, offering improved therapeutic efficacy but with risks such as immunosuppression and opportunistic infections.

**Patient concerns:**

We report a case of a patient diagnosed with DLBCL who experienced immunosuppression as a result of treatment with rituximab and a Bruton’s tyrosine kinase inhibitor, which subsequently led to Strongyloides stercoralis infection.

**Diagnoses:**

The patient was diagnosed with S. stercoralis infection, confirmed by appropriate diagnostic tests after the onset of clinical symptoms suggestive of parasitic infection.

**Interventions:**

The patient was treated with a combination of rituximab and a Bruton’s tyrosine kinase inhibitor as part of her DLBCL therapy. Antiparasitic treatment was started after diagnosis of S. stercoralis infection.

**Outcomes:**

The patient’s infection was successfully managed with antiparasitic therapy, although the case highlights the need for vigilant monitoring of immunosuppressive therapy in patients with DLBCL due to the risk of opportunistic infections.

**Lessons:**

This case highlights the potential complications of targeted therapies in DLBCL, particularly the risk of opportunistic infections such as S. stercoralis. It highlights the importance of careful patient monitoring and prompt intervention to effectively manage such infections.

## 1. Introduction

Diffuse large B-cell lymphoma (DLBCL) is the most common subtype of adult lymphoma and is characterized by its aggressive and heterogeneous nature. Although the combination of rituximab with the CHOP regimen has improved outcomes for DLBCL patients, approximately one third of patients still experience relapse or refractory disease.^[1]^ In recent years, advances in next-generation sequencing technology have led to more precise classification of DLBCL. This has facilitated the development of new targeted therapies for DLBCL-related pathways and molecular targets, including immunosuppressive agents, Bruton’s tyrosine kinase (BTK) inhibitors and chimeric antigen receptor T-cell products, which offer new treatment options.^[2–4]^ However, while new drugs are improving patient outcomes, infections are a significant problem and the incidence of opportunistic infections after immunosuppression is increasing. Viral infections after the use of rituximab and fungal infections after the use of regimens containing BTK inhibitors have been reported in the literature.^[5,6]^ There are no reports of immunodeficiency leading to Strongyloides stercoralis infection after combined targeted therapy with rituximab and BTK inhibitors. We present a case report of S. stercoralis infection in a patient with DLBCL involving the central nervous system who received targeted therapy to explore the pathogenesis, diagnosis and treatment of opportunistic infections in this immunocompromised state.

## 2. Case report

A 74-year-old man was admitted on March 26, 2021 because of “blurred vision and mood disturbance for 1 month.” His medical history includes hypertension, hypertensive heart disease and severe chronic obstructive pulmonary disease. On admission, physical examination revealed multiple enlarged lymph nodes in the neck, axilla and groin, the largest measuring approximately 4 cm × 1.5 cm. These nodes were moderately firm, well-demarcated, relatively firm and nontender, and not adherent to the surrounding tissues. The overlying skin showed no signs of congestion or ulceration. Blood routine showed leukocytes 18.21 × 10^9^/L, neutrophils 82.2%, hemoglobin 127 g/L, platelets 287 × 10^9^/L. Cerebrospinal fluid routine and biochemistry were normal and no abnormal cells were detected. Superficial lymph node ultrasonography showed multiple lymph node enlargement in the neck, axilla and groin. Cranial magnetic resonance imaging revealed the presence of anomalous signals in the bilateral basal ganglia, the right paraventricular region, and the optic tract. Bone marrow pathology revealed no evidence of lymphoma cell involvement. To elucidate the diagnosis, a biopsy of the right neck mass and resection of the left oropharyngeal mass were conducted on March 16, 2021. The postoperative pathology indicated the presence of DLBCL. In order to ascertain the stage of the disease, a positron emission tomography scan was conducted, which revealed the presence of multiple enlarged lymph nodes in the left posterior oropharyngeal wall, right neck, left axilla, bilateral pelvic vessels and bilateral inguinal region, exhibiting increased metabolism, indicative of lymphoma infiltration.

The patient was diagnosed with DLBCL (non-growth center type, stage IVA, International Prognostic Index score 4, high-risk group), with involvement of the oropharynx and central nervous system. In view of the patient’s advanced age, comorbid hypertension, hypertensive heart disease, very severe chronic obstructive pulmonary disease, high risk of chemotherapy, and the refusal of chemotherapy by the patient and his family, immune-targeted therapy was selected as the treatment option. On April 1, 2021, the patient started the orelabrutinib, rituximab, lenalidomide (OR2) regimen consisting of rituximab at a dose of 375 mg per square meter of body surface area on day 0, lenalidomide at a dose of 15 mg per day on days 1 to 21, and obinutuzumab at a dose of 150 mg per day. Following 8 cycles of treatment with the OR2 regimen, a positron emission tomography scan demonstrated a complete remission (CR). Subsequently, the patient underwent the 9th cycle of OR2 regimen chemotherapy on February 15, 2022.

**Figure 1.**
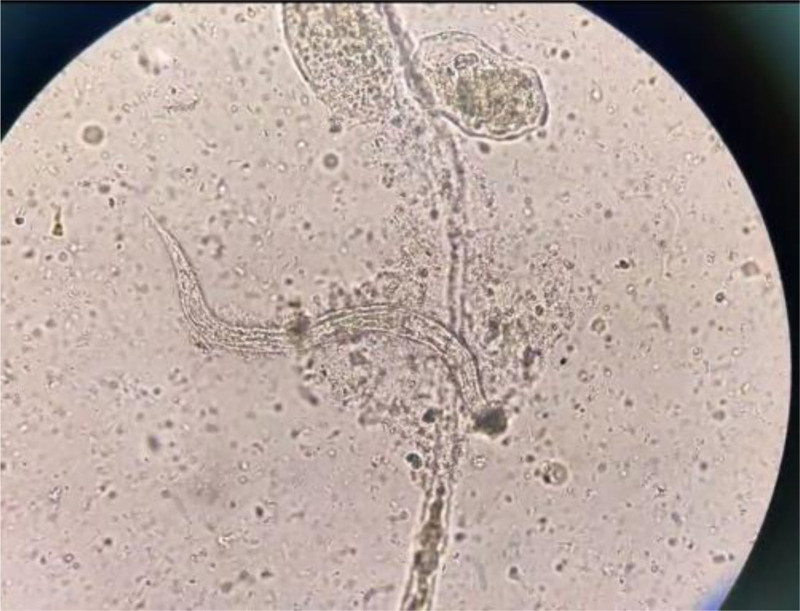
Larvae of Strongyloides stercoralis (OLYMPUS CX22LED,×100).

**Figure 2.**
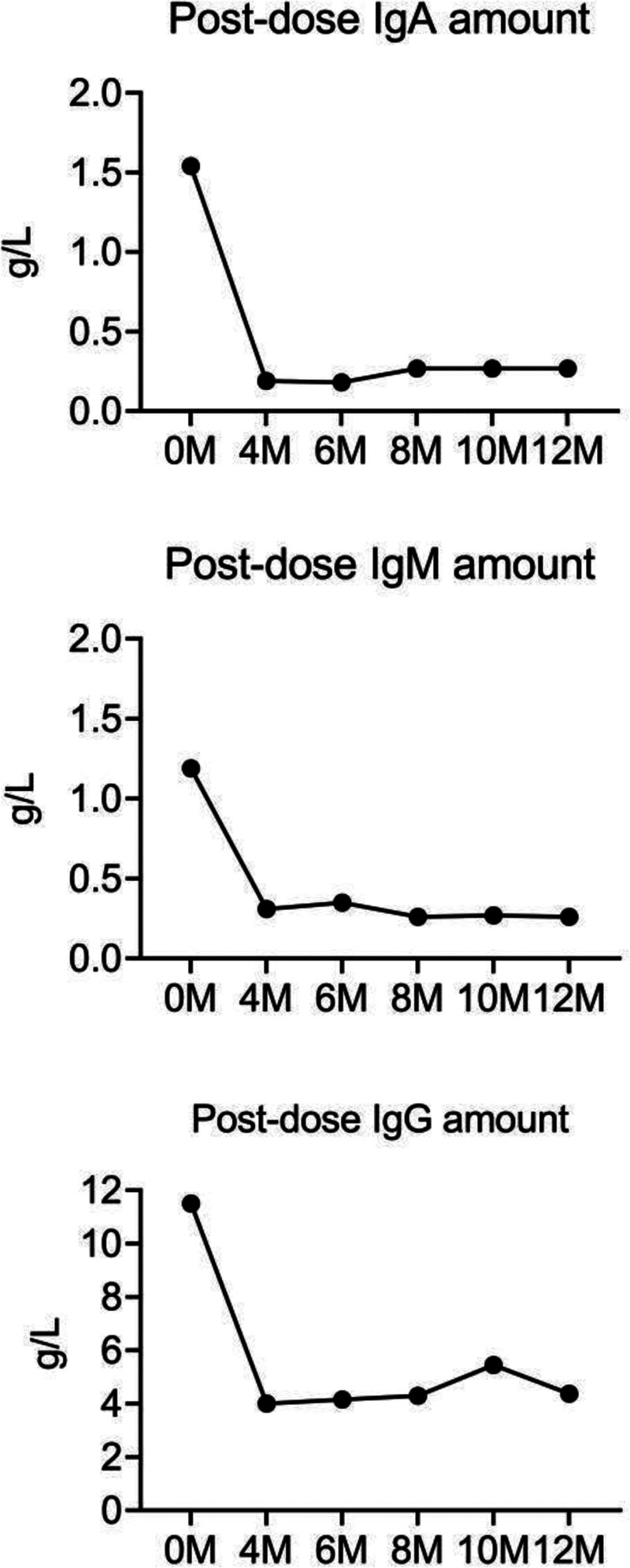
Changes of humoral immune function before and after treatment (IgA, IgM, IgG). IgA = immunoglobulin A, IgG = immunoglobulin G, IgM = immunoglobulin M.

On March 14, 2022, the patient was admitted to the hospital with a diagnosis of recurrent diarrhea. Prior to admission, the patient exhibited pruritus and elevated eosinophil levels, with a poor response to anti-allergic treatment. A comprehensive examination of the patient’s stool samples revealed the presence of S. stercoralis larvae (Fig. 1; see Video S1, Supplemental Digital Content, http://links.lww.com/MD/O418, under the microscope, surviving larvae of S. stercoralis can be observed). A review of the patient’s humoral immune function before and after treatment revealed a progressive decrease in immunoglobulin levels (immunoglobulin A [IgA], immunoglobulin G [IgG] and immunoglobulin M [IgM]) following immunotherapy (Fig. 2). Additionally, T-cell immunity indicated reduced absolute counts of CD4, CD8 and CD3 (Table 1). In light of the patient’s deficiencies in both humoral and cellular immunity following targeted therapy, immunosuppression ultimately resulted in the development of a parasitic infection. On March 16, 2022, the patient was treated with albendazole (400 mg/day for 3 days), resulting in an improvement in diarrhea and overall condition. The patient was discharged on March 22, 2022, and subsequently began maintenance therapy with lenalidomide (15 mg/day, days 1–21, every 28 days per cycle). Unfortunately, the patient passed away from COVID-19 in December 202f2.

**Table 1:** T cell immunity.

Analysis project	Analysis results	Normal reference value	Judgment
CD4 count	140	500–1440	Low
CD8 count	88	238–1250	Low
CD3 count	240	770–2860	Low
CD4/CD8	1.59	1.0–2.47	Normal

CD3= cluster of differentiation 3 (T cell co-receptor), CD4 = cluster of differentiation 4 (T-helper cells), CD8 = cluster of differentiation 8 (cytotoxic T cells).

## 3. Discussion

In recent years, the treatment of lymphoma has changed significantly from traditional nonspecific anti-proliferative chemotherapy to targeted therapy of specific molecular signaling pathways combined with traditional chemotherapy. Monoclonal antibodies, protease inhibitors and immunomodulatory drugs play an important role in the treatment of lymphoma.^[7]^ With the in-depth study of lymphatic tumor signaling pathway inhibitors, drugs such as Bruton’s tyrosine kinase are also providing new treatment ideas. Tyrosine kinase is the central mediator of B cell receptor signaling and is required for normal B cell development.^[8]^ In vitro and in vivo experiments show that BTK inhibitors are effective against lymphoid tumors such as chronic lymphocytic leukemia, mantle cell lymphoma, follicular lymphoma, DLBCL and multiple myeloma.^[9,10]^ BTK inhibitors can cross the blood-brain barrier and are well distributed in the central nervous system (CNS), where they can effectively inhibit B-cell proliferation and promote apoptosis.^[8]^ In a phase II study evaluating the efficacy and safety of ibrutinib in relapsed or refractory CNS lymphoma, 23/31 (74%) of patients with primary CNS lymphoma and 9/15 (60%) of patients with secondary CNS lymphoma achieved clinical remission, with 12 and 7 CRs, respectively. This study confirmed the single-agent efficacy of ibrutinib in relapsed/refractory CNS lymphoma.^[11]^ In this case, the patient presented with lymphoma involving the oropharynx and central nervous system, was of advanced age, and had a variety of underlying medical conditions. The patient’s general condition was poor, and he was unable to tolerate chemotherapy. Accordingly, the OR2 regimen was selected for the treatment plan. At the follow-up appointment, the patient was found to be in CR, indicating that the treatment was successful.

But while targeted therapy brings efficacy, infection is a problem we cannot ignore. Rituximab is a monoclonal antibody that specifically binds to the transmembrane protein CD20 to trigger an immune response that mediates B-cell lysis and kills B-cells. Another targeted agent, Bruton’s tyrosine kinase,^[12]^ is expressed primarily in immature B cells and myeloid cells. As a key regulator of the B cell receptor signaling pathway, BTK can regulate the maturation and differentiation of normal B cells. BTK deficiency blocks the transformation of progenitor B cells into pre-B cells, mature B cells and inhibits the production of mature B cells, leading to immunodeficiency diseases. The examination of our patient also shows that the body is in a state of immunodeficiency. The function of immunoglobulin IgA, IgG, IgM, CD4, CD8 is reduced.

The incidence of opportunistic infections also increases in immunocompromised states, with 39% of patients in the study by Lionakis et al^[13]^ developing invasive fungal infections, and there are also studies suggesting that serious infections (including fatal bacterial, fungal and new or reactivated viral infections) may occur during and after completion of rituximab-based therapy. The literature has documented cases of fungal and viral infections, but there have been no reports of parasitic infections following targeted therapy with the combination of rituximab and BTK inhibitors.

S. stercoralis is a life-threatening parasitic infection, particularly in immunosuppressed patients, and death usually occurs within a few days. The disease has a worldwide distribution and is endemic in tropical and subtropical regions.^[14]^ The clinical manifestations of acute infection are mainly nonspecific respiratory and gastrointestinal symptoms, usually accompanied by blood eosinophilia. The majority of patients with chronic S. stercoralis infection present only with increased blood eosinophilia, and some patients develop a rash or mild gastrointestinal symptoms.^[15,16]^ In previous literature, patients infected with parasites are often those who have been using corticosteroids for a long period,^[17]^ and there have also been case reports of hematologic malignancies complicated by S. stercoralis infection. A 9-year-old girl presented with localized bone pain and eosinophilia, and was diagnosed with acute lymphoblastic leukemia (ALL) along with a concurrent S. stercoralis infection.^[18]^ The patient underwent anti-parasitic treatment before chemotherapy. Furthermore, a retrospective study at MD Anderson Cancer Center found that the incidence of S. stercoralis infection in cancer patients was approximately 1 per 10,000 new cancer cases, primarily in those with solid organ malignancies.^[19]^ However, there are no reports of S. stercoralis infection following lymphoma targeted therapy. In this case, the patient presented with pruritus, elevated eosinophils and poor anti-allergic treatment, then developed recurrent diarrhea, and fecal microscopy revealed surviving S. stercoralis, and his condition improved after deworming treatment. The patient was considered immunocompromised after treatment with targeted drugs, and parasitic infections occurred.

The treatment of DLBCL is changing every day. While achieving the curative effect, we cannot ignore the side effects. In patients with recurrent diarrhea with rash/itching and eosinophilia, we need to be aware of intestinal parasitic infection: we need to do multiple stool microscopy to identify the source of infection as soon as possible and treat it in time. At the same time, we need to reinforce personal hygiene and clean eating.

## Author contributions

**Methodology:** Lili Zeng.

**Visualization:** Nina Cai.

**Writing – original draft:** Wanyi Liu, Zechuan Wang, Huiqiang Wu.

**Writing – review & editing:** Weihuang Zhuang, Jianxin Guo.

## Supplementary Material


